# Sonographic evaluation of the shoulder in asymptomatic elderly subjects with diabetes

**DOI:** 10.1186/1471-2474-11-278

**Published:** 2010-12-07

**Authors:** Michele Abate, Cosima Schiavone, Vincenzo Salini

**Affiliations:** 1 Department of Neuroscience and Imaging, Infrared Imaging Laboratory, Institute of Advanced Biomedical Technologies (ITAB), "University G. d' Annunzio" Chieti - Pescara, Via dei Vestini 31, 66013, Chieti Scalo (CH), Italy; 2 Echography Unit, Department of Medicine and Sciences of Aging, University "G. d' Annunzio" Chieti - Pescara, Via dei Vestini 31, 66013, Chieti Scalo (CH), Italy; 3 Department of Human Movement Science, University "G. d 'Annunzio" Chieti - Pescara, Via dei Vestini 31, 66013, Chieti Scalo (CH), Italy

## Abstract

**Background:**

The prevalence of rotator cuff tears increases with age and several studies have shown that diabetes is associated with symptomatic shoulder pathologies. Aim of our research was to evaluate the prevalence of shoulder lesions in a population of asymptomatic elderly subjects, normal and with non insulin - dependent diabetes mellitus.

**Methods:**

The study was performed on 48 subjects with diabetes and 32 controls (mean age: 71.5 ± 4.8 and 70.7 ± 4.5, respectively), who did not complain shoulder pain or dysfunction. An ultrasound examination was performed on both shoulders according to a standard protocol, utilizing multiplanar scans.

**Results:**

Tendons thickness was greater in diabetics than in controls (Supraspinatus Tendon: 6.2 ± 0.09 mm *vs *5.2 ± 0.7 mm, p < 0.001; Biceps Tendon: 4 ± 0.8 mm *vs *3.2 ± 0.4 mm, p < 0.001). Sonographic appearances of degenerative features in the rotator cuff and biceps were more frequently observed in diabetics (Supraspinatus Tendon: 42.7% *vs *20.3%, p < 0.003; Biceps Tendon: 27% *vs *7.8%, p < 0.002).

Subjects with diabetes exhibited more tears in the Supraspinatus Tendon (Minor tears: 15 (15.8%) *vs *2 (3.1%), p < 0.03; Major tears: 15 (15.8%) *vs *5 (7.8%), p = ns), but not in the long head of Biceps. More effusions in subacromial bursa were observed in diabetics (23.9% *vs *10.9%, p < 0.03) as well as tenosynovitis in biceps tendon (33.3% *vs *10.9%, p < 0.001).

In both groups, pathological findings were prevalent on the dominant side, but no difference related to duration of diabetes was found.

**Conclusions:**

Our results suggest that age - related rotator cuff tendon degenerative changes are more common in diabetics.

Ultrasound is an useful tool for discovering in pre - symptomatic stages the subjects that may undergo shoulder symptomatic pathologies.

## Background

Several sonographic studies, performed in the general population, show that the prevalence of rotator cuff tears increases with age. The prevalence of tears ranges widely, roughly from 0 - 15% in the 60 s to 30 - 50% in 80 s, these differences being explained by characteristics of subjects enrolled and sonographic criteria by which lesions were identified [1-9].

Magnetic Resonance Imaging is a more sensitive methodology than ultrasound (US) in detecting pathological changes in asymptomatic shoulders. However, its use for epidemiological purposes is limited by higher costs and lower availability [10,11].

Moreover, it is well known that diabetes is a strong risk factor for rotator cuff pathologies, as shown by studies performed in symptomatic subjects [12-22]. In addition, after a surgical repair, diabetics show a restricted shoulder range of motion [23]and a higher incidence in re - tears[24].

However, to our knowledge, there are no studies, which evaluated asymptomatic elderly subjects with diabetes. Therefore, it could be of interest to investigate whether diabetes has an additive effect on age - related tendon degeneration and whether US evaluation of the shoulder in pre - symptomatic stage could be a useful tool for discovering subjects at risk.

In addition, it must be considered that the majority of sonographic studies has been focused on supraspinatus tendon tears [1,2,5-9], whereas less attention has been paid to other tendons of rotator cuff and anatomical structures of the shoulder [3,25].

Therefore, aim of this study was twofold: first, to evaluate the prevalence of sonographic shoulder lesions in asymptomatic elderly subjects, normal and diabetics; second, to describe, beside supraspinatus tears, other abnormalities of anatomical structures of clinical interest which could occur in these subjects.

## Methods

### Subjects

All the subjects enrolled in the study were recruited from the Outpatients Service of the Medicine and Science of Aging Department of Chieti - Pescara University.

Inclusion criteria were the following: 1) living independently in the community; 2) age > 65 years; 3) right - handedness; 4) absence of pain or acceptable discomfort in the shoulder joint, spontaneous or during usual activities of daily living; 5) no subjective dysfunction; 6) no history of trauma or surgery of the shoulder joint.

Patients with rheumatic disorders, endocrinopathies, malignancies and systemic diseases (renal, hepatic, cardiac, etc.), treated with steroids or NSAID, were excluded.

The local Ethics Committee approved the study design and informed written consent was obtained from all the patients.

The study group included 48 subjects with non insulin - dependent diabetes mellitus (NIDDM). The diagnosis of NIDDM was based on American Diabetes Association criteria [26].

The control group was made by 32 subjects, matched for age and sex, but without NIDDM, and selected with the same inclusion/exclusion criteria.

The age of onset of diabetes, current therapies and comorbidities were registered.

Hypertension was diagnosed when the subjects were taking antihypertensive drugs, Coronary Arterial Disease when they suffered from angina or myocardial infarction and Peripheral Arterial Disease when Ankle Brachial Index was less than 0.90 mmHg.

Subjects were also classified according their previous working activities, sports and hobbies. Home and office work was considered as light work; farm, factory and building industry work was considered heavy work.

### Ultrasound evaluation

US examination was performed by the same operator (AM), by means of a multi - frequency (5 - 14 MHz) linear array probe. The examiner was not blinded to the clinical status of the subjects.

Both shoulders were evaluated, according to a standard protocol, previously described by Papatheodorou et al. [27].

The following tendons were evaluated: Supraspinatus (SST), Infraspinatus (IST), Subscapularis (SBT) and long head of the Biceps (BT). In addition, the subacromial - subdeltoid (SAD) bursa was studied.

Maximal SST thickness was measured on a longitudinal view, just in front of the lateral part of the humeral head [27]; the thickness of the long head of BT was measured into the bicipital groove [28].

The presence of dishomogeneous hypo- or hyperechoic thicknening, diffuse or focal, of the tendon, associated with loss of the normal fibrillar pattern and/or irregularity of the tendon margins, was interpreted as sign of degeneration.

In addition, but only in BT, which is provided of a synovial sheat, the appearance of an anechoic area around the tendon, associated or not with synovial proliferation, was considered as a sign of tenosynovitis [29].

SST, IST and SBT tears were classified as follows:

1) Partial thickness tear: focal hypoechoic discontinuity with irregular margins at the bursal or articular side or located intratendinously. A bursal - side tear produces flattening of the bursal surface, with loss of the superior convexity of the tendon, while an articular - side tear appears as a distinct hypoechoic or mixed hyper - hypoechoic defect of the articular surface, abutting the articular cartilage [27].

2) Full thickness tear: full defect in the tendon from the bursal to the articular margin. Hypoechoic fluid may fill the tear, with loss of the normal outward convexity of the tendon at this site. Moreover, owing to the pressure applied with the transducer, the deltoid muscle can abut against the humeral head [27].

Taken into consideration the distance between the ends of the tendon, tears were divided in small ( < 1 cm), large (more than 1 but less than 3 cm) or massive (> 3 cm).

For simplicity sake, we considered a) Minor tears ( including partial thickness tears and small full thickness ruptures) and b) Major tears (including large full thickness tears and massive ruptures).

For BT, a partial tear was reported as a focal hypoechoic discontinuity, with irregular borders, located intratendinously, while the non visualization of the tendon into the bicipital groove, with associated bulbous appearance of the biceps muscle, was reported as complete tear [27].

Involvement of SAD was identified when accumulation of anechoic fluid, with or without hypoechoic swelling of the synovia, appeared within it; it was graded subjectively as normal (distension < 1 mm), slightly increased (1 - 2 mm) or clearly increased (> 2 mm).

### Data analysis

Data are reported as mean and standard deviation (mean ± SD) for continuous variables, whereas categorical and dichotomous variables are reported as frequencies and percentage. The tendon thickness and the percentage of US abnormalities found in the shoulders of diabetic subjects were compared with that observed in controls. Afterwards, the differences in tendon thickness between dominant and non - dominant side and according to diabetes duration were analyzed.

The significance level was determined at p < 0.05. The two - sample Student's *t - *test was used to compare continuous variables when the distribution of data was normal; the Wilcoxon's rank sum test was used otherwise. The χ _2 _test was used to evaluate associations between categorical data.

All analyses were done using SAS statistical software, release 8.1.

## Results

Demographic and clinical characteristics of diabetic and control subjects are presented in Table 1.

**Table 1:** Demography and associated diseases

	Controls	Diabetics	p
**Age**	70.7 ± 4.5 (65 - 82)	71.5 ± 4.8 (65 - 84)	ns

**M : F**	20 : 12	30 : 18	ns

**Previous working activity**			
- Heavy work	10 (31.25%)	12 (25%)	Ns
- Light work	22 (68.75%)	36 (75%)	ns

**Hypertension**	10 (31.2%)	29 (60.4%)	**0.001**

**Coronary Heart Disease**	6 (18.7%)	18 (37.5%)	**0.007**

**Peripheral Artery Disease**	5 (15.6%)	12 (25%)	ns

The subjects did not differ for age, sex and previous working activity. None of the subjects enrolled practised sports or had hobbies involving the use of shoulder joints. Cardiovascular complications (Hypertension, Coronary Heart Disease), as expected, were observed more frequently in diabetics.

The duration of diabetes was less than 10 years (mean 6.8 ± 1.9) in 34 subjects and more than 10 years (mean 14.7 ± 2.2 years) in 14. In all the participants, HbA1c levels were < 8.0 gr/dl.

**Figure 1:**
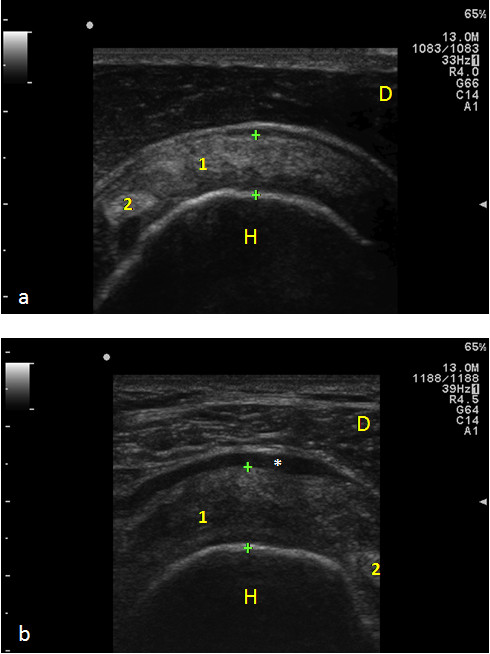
**Ultrasound appearance of normal and degenerated Supraspinatus Tendon**. Panel a) Transverse scan: a normal fibrillar pattern and thickness of Supraspinatus Tendon is observed (between calipers). Panel b) Supraspinatus Tendon is thickened (calipers), hypoechoic, dishomogeneous, with loss of the normal fibrillar pattern. In this picture, an effusion in the subacromial bursa is present (*). 1) Supraspinatus tendon; 2) Biceps tendon; H) Humeral head; D) Deltoid muscle.

SST and BT thickness was significantly greater in diabetics than in controls both in the dominant and non dominant side (Figure 1; Table 2).

**Table 2:** Sonographic findings in diabetics and control subjects

	DOMINANT SIDE			NON DOMINANT SIDE			BOTH SHOULDERS		
	
	Controls	Diabetics	p	Controls	Diabetics	p	Controls	Diabetics	p
**SST**									
Thickness (mm)	5.2 ± 0.7	6.2 ± 0.9	**0.001**	4.7 ± 0.6	5.6 ± 0.6	0.08			
Minor lesions	1 (3.1%)	10 (20.8%)	**0.02**	1 (3.1%)	5 (10.4%)	0.22	2 (3.1%)	15 (15.8%)	**0.03**
Major lesions	3 (9.3%)	10 (20.8%)	0.17	2 (6.2%)	5 (10.4%)	0.5	5 (7.8%)	15 (15.8%)	0.14

**BT**									
Thickness (mm)	3.2 ± 0.4	4 ± 0.8	**0.001**	3 ± 0.4	3.4 ± 0.5	**0.001**			
Minor lesions	1 (3.1%)	2 (4.1%)	0.06	1 (3.1%)	1 (2%)	0.09	2 (3.1%)	3 (3.1%)	0.00
Major lesions	1 (3.1%)	3 (6.2%)	0.39	1 (3.1%)	2 (4.1%)	0.06	2 (3.1%)	5 (5.1%)	0.37

**Degeneration**									
Rotator cuff	7 (21.8%)	22 (45.8%)	**0.02**	6 (18.7%)	19 (39.5%)	**0.04**	13 (20.3%)	41 (42.7%)	**0.003**
BT	3 (9.3%)	17 (35.4%)	**0.008**	2 (6.2%)	9 (28.1%)	0.11	5 (7.8%)	26 (27.8%)	**0.002**

**Effusion**									
SAD	4 (12.5%)	16 (33.3%)	**0.03**	3 (9.3%)	7 (14.5%)	**0.4**	7 (10.9%)	23 (23.9%)	**0.03**
BT	5 (15.6%)	21 (43.7%)	**0.008**	2 (6.2%)	11 (22.9%)	**0.04**	7 (10.9%)	32 (33.3%)	**0.001**

**Figure 2:**
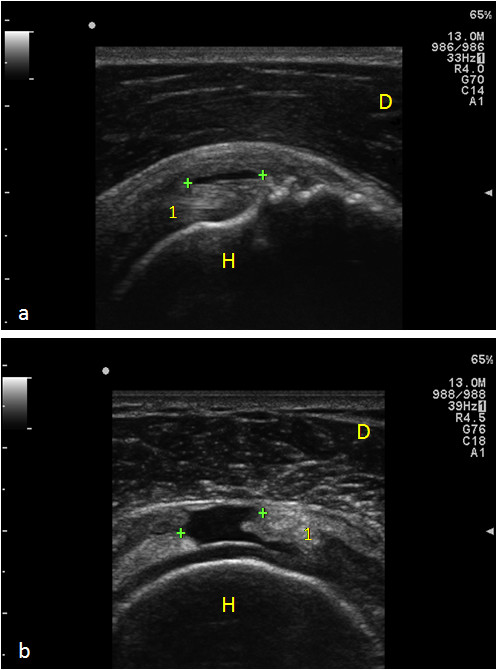
**Supraspinatus Tendon tears**. Panel a) Longitudinal scan: an intratendinous partial thickness tear is reported as focal hypoechoic discontinuity (calipers) with irregular margins. Panel b) Transverse scan: a full defect in the tendon from the bursal to the articular margin, filled with anechoic fluid, is observed (calipers). 1) Supraspinatus tendon; H) Humeral head; D) Deltoid muscle.

In both groups, tears of SST were more frequently observed (Figure 2). The percentage of both minor and major tears was higher in diabetics, but the difference was significant only for SST minor tears. Massive tears of SST were always associated with an involvement of IFT. No tears in the SBT were registered.

Subjects with diabetes duration more than 10 years exhibited more SST and BT tears, but the difference was not significant at statistical analysis: 1) SST: Minor tears 7/28 (25%) *vs *8/68 (11.7%), p = 0.10; Major tears 6/28 (21.4%) *vs *9/68 (13.2%), p = 0.31; 2) BT: Minor tears 1/28 (3.5%) vs 2/68 (2.9%), p = 0.87; Major tears 3/28 (10.7%) vs 2/68 (2.9%), p = 0.11).

The prevalence of degenerative abnormalities in rotator cuff tendons and BT was significantly higher in diabetics.

**Figure 3:**
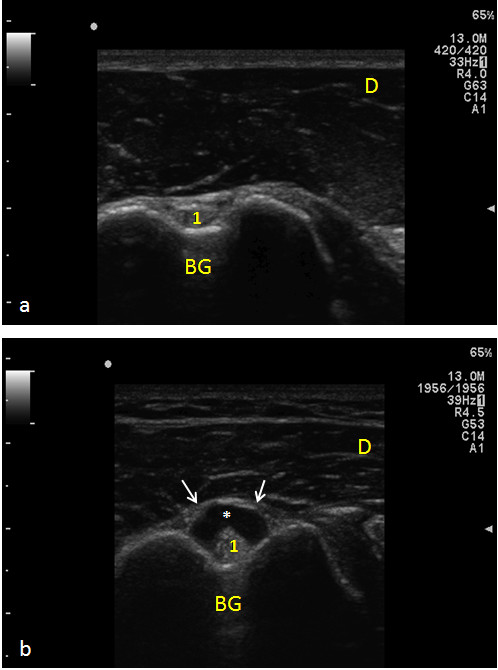
**Tenosynovitis of the Biceps tendon**. Panel a) Biceps tendon into the bicipital groove (transverse scan): the fibrillar pattern and thickness are normal. Panel b) Fluid around the tendon (*) expands the synovial sheet (arrows) expression of tenosynovitis. A mild thickening of Biceps tendon is observed. 1) Biceps tendon; BG) Bicipital groove; D) Deltoid muscle.

Tenosynovitis of the long head of the BT and SAD bursitis were more frequent in diabetics (Figure 3; Table 2).

## Discussion

Several studies have shown that the prevalence of rotator cuff tendons tears is increased in elderly subjects, with or without shoulder pain or movement limitation [1-9]. Moreover, it is well known that patients with diabetes are at increased risk for shoulder pathologies, such as frozen shoulder or rotator cuff tears [12-22]. In addition, after a surgical repair, diabetics show a restricted range of motion of the shoulder [23] and a higher incidence in re-tears. This observation can be related to the intrinsic poor quality of the tissue that is being repaired [24].

The results of our study suggest that, also in asymptomatic subjects, age - related rotator cuff tendon changes are more common in diabetics. This conclusion is supported by the observation of a higher prevalence of tears and of degenerative phenomena in diabetics, as well as by the increased thickness of supraspinatus and biceps tendons, which is due to the abnormal storage of collagen layers in the tissue and, therefore, is itself an expression of degenerative changes [20].

These observations are of clinical relevance, because it has been shown by Yamaguchi et al. [30,31], in a 2.8 years follow - up study, that pain and limitation in functional ability can develop in a large percentage (50%) of people with asymptomatic tears at baseline.

Besides tendinopathic degenerative lesions, bursal and peritendinous effusions were observed, which could be an expression of early reactive inflammation to minimal tendon tears, following minor and unrecognized traumatic events [32-36].

Another clinical observation coming from our study is the increased prevalence of pathological findings in the dominant side, that confirms the theory that overuse may have a significant pathogenetic role [9,37,38].

However, due to the difficulty of getting definite information about working - related lesions, we have not been able to differentiate work - related injuries versus intrinsic diabetes - induced changes in the rotator cuff.

No significant correlation was observed with the duration of diabetes. The lack of association may be explained by the difficulty in establishing the age of diabetes onset. Indeed, subjects could have glucose intolerance or mild NIDDM for a significant period of time before the clinical diagnosis of diabetes.

From the pathogenetic point of view, it is likely that, in individuals with no history of significant trauma, rotator cuff tears are mainly caused by intrinsic degenerative changes, related to aging, vascular factors or mechanical impingement [6,34,36,39,40].

The biochemical mechanisms of tendon degeneration are similar in ageing and diabetes.

The most important abnormality is the non - enzymatic glycosylation of collagen with advanced glycation end products (AGEs) formation [41-43].

The spontaneous condensation of glucose and metabolic intermediates (e.g. triose phosphates, glyoxal and methylglyoxal) with free aminogroups in lysine, hydroxylysine or arginine leads to a covalent bond between the sugar and the aminoacid (the Amadori product), and subsequent reactions give rise to the formation of AGEs.

AGEs affect physical and chemical properties of proteins, increasing the amount of intermolecular collagen cross - links. This results in reduction of the solubility of collagen that gets tougher, stiffer and weaker, loses its elasticity and is more likely to tear [44].

Another mechanism, by which AGEs may exert noxious effects, is represented by their action on specific receptors (RAGE), which have been identified on the membrane of chondrocytes, tenocytes and fibroblasts [45,46].

Ligand engagement of RAGE triggers cell - specific signalling, resulting in enhanced generation of reactive oxygen species, and in the activation of the transcription of nuclear factor NF - kB [47,48]. This, in turn, accelerates AGEs cross - link formation in collagen fibers [49,50] and leads to sustained upregulation of pro - inflammatory mediators, adhesion molecules and to a dysfunctional cell phenotype [48,51,52].

In diabetes RAGE, Vascular Endothelial Growth Factor and Cytokines are overexpressed [53-56] and it may explain why diabetics show increased prevalence of lesions and inflammatory reactions.

Beside the AGE - mediated pathogenetic mechanism, microvascular disease may lead to tissue hypoxia, resulting in production of oxygen free radicals, which, in turn, leads to overproduction of growth factors and cytokines [57-59].

Some limitations of this study must be acknowledged. Pain and functional limitation were evaluated on a self report basis, which is highly subjective. Active and passive ranges of motion of the shoulder were not measured. As matter of fact, functional impairment or pain in the extreme degrees of movements could be present in these subjects, who were yet able in performing usual activities of daily living, but who could avoid, spontaneously or unconsciously, some disturbing tasks. Spurs or other bone abnormalities were not taken into account in this study, which was mainly aimed at tendon evaluation. Therefore, we cannot state whether individuals with diabetes had a higher prevalence of lesions that could be treated surgically to prevent impingement type tears. Moreover, the examiner was not blind to whether or not the individuals had diabetes or not.

Finally, it must be acknowledged that US investigation has limited reliability in detecting partial thickness tears and intra - articular tears of biceps tendon [7,60,61].

## Conclusions

Our results demonstrate that NIDDM worsen the tendon degeneration in aged subjects. US imaging, beside clinical evaluation, is an useful tool for discovering in pre - symptomatic stages the subjects at risk, who may undergo to shoulder pathologies. In these subjects, that represent a growing segment of elderly population, a careful metabolic control by means of diet and anti - diabetics drugs is recommended and the progression of tear size should be monitored over time.

## List of abbreviations

**NIDDM**: Non Insulin Dependent Diabetes Mellitus; **US**: Ultrasound; **SST**: Supraspinatus; **IST**: Infraspinatus; **SBT**: Subscapularis; **BT**: Long head of the biceps tendon; **SAD**: Subacromial subdeltoid bursa; **AGE**: Advanced glycation end product; **RAGE**: Receptor advanced glycation end product; **PGE2**: Prostaglandin E2; **NO**: Nitric Oxide

## Competing interests

The authors declare that they have no competing interests.

## Authors' contributions

AM: Planning of the study. Echographic evaluation. Writing the paper; CS: Interpretation of US findings and bibliographic research; VS: Supervision of the study and of the final version of the paper.

All authors read and approved the final manuscript.

## Pre-publication history

The pre-publication history for this paper can be accessed here:

http://www.biomedcentral.com/1471-2474/11/278/prepub
